# Soluble CD4 and CD4-Mimetic Compounds Inhibit HIV-1 Infection by Induction of a Short-Lived Activated State

**DOI:** 10.1371/journal.ppat.1000360

**Published:** 2009-04-03

**Authors:** Hillel Haim, Zhihai Si, Navid Madani, Liping Wang, Joel R. Courter, Amy Princiotto, Aemro Kassa, Marciella DeGrace, Kathleen McGee-Estrada, Megan Mefford, Dana Gabuzda, Amos B. Smith, Joseph Sodroski

**Affiliations:** 1 Department of Cancer Immunology and AIDS, Dana-Farber Cancer Institute, Division of AIDS, Harvard Medical School, Boston, Massachusetts, United States of America; 2 Department of Chemistry, University of Pennsylvania, Philadelphia, Pennsylvania, United States of America; 3 Department of Immunology and Infectious Diseases, Harvard School of Public Health, Boston, Massachusetts, United States of America; Northwestern University, United States of America

## Abstract

Binding to the CD4 receptor induces conformational changes in the human immunodeficiency virus (HIV-1) gp120 exterior envelope glycoprotein. These changes allow gp120 to bind the coreceptor, either CCR5 or CXCR4, and prime the gp41 transmembrane envelope glycoprotein to mediate virus–cell membrane fusion and virus entry. Soluble forms of CD4 (sCD4) and small-molecule CD4 mimics (here exemplified by JRC-II-191) also induce these conformational changes in the HIV-1 envelope glycoproteins, but typically inhibit HIV-1 entry into CD4-expressing cells. To investigate the mechanism of inhibition, we monitored at high temporal resolution inhibitor-induced changes in the conformation and functional competence of the HIV-1 envelope glycoproteins that immediately follow engagement of the soluble CD4 mimics. Both sCD4 and JRC-II-191 efficiently activated the envelope glycoproteins to mediate infection of cells lacking CD4, in a manner dependent on coreceptor affinity and density. This activated state, however, was transient and was followed by spontaneous and apparently irreversible changes of conformation and by loss of functional competence. The longevity of the activated intermediate depended on temperature and the particular HIV-1 strain, but was indistinguishable for sCD4 and JRC-II-191; by contrast, the activated intermediate induced by cell-surface CD4 was relatively long-lived. The inactivating effects of these activation-based inhibitors predominantly affected cell-free virus, whereas virus that was prebound to the target cell surface was mainly activated, infecting the cells even at high concentrations of the CD4 analogue. These results demonstrate the ability of soluble CD4 mimics to inactivate HIV-1 by prematurely triggering active but transient intermediate states of the envelope glycoproteins. This novel strategy for inhibition may be generally applicable to high–potential-energy viral entry machines that are normally activated by receptor binding.

## Author Summary

Human immunodeficiency virus type 1 (HIV-1) is the cause of the global AIDS epidemic. HIV-1 gains entry into its target cells by fusing with the cell membrane, a process that begins with the interaction of the viral envelope glycoproteins with cell-surface receptors. HIV-1 uses two receptors on the target cell: CD4 and CCR5/CXCR4. Binding of the virus to the primary receptor, CD4, primes the viral envelope glycoproteins to mediate the fusion of the viral membrane and the membrane of the target cell. Soluble forms of the CD4 receptor and small molecules that mimic the effects of CD4 can inhibit virus infection; however, how this inhibition occurs is still unknown. In this report, we show that soluble mimics of CD4 inhibit HIV-1 infection by prematurely triggering the viral envelope glycoproteins. The unstable activated state of the virus lasts only a few minutes, after which the virus loses the ability to infect cells. This novel strategy for inhibition may be generally applicable to other viruses besides HIV-1, some of which are also activated by binding to their receptors.

## Introduction

The entry of human immunodeficiency virus type 1 (HIV-1) into target cells is mediated by the trimeric envelope glycoprotein complex, which consists of three gp120 exterior envelope glycoproteins and three gp41 transmembrane envelope glycoproteins [1]. Binding of gp120 to the receptor, CD4, on the target cell surface induces major conformational changes in the envelope glycoproteins [2]. These changes allow gp120 to bind the viral coreceptor, either CXCR4 or CCR5 [3]–[7]. CD4 binding also induces the formation of a gp41 pre-hairpin intermediate, in which three hydrophobic grooves on the surface of a coiled coil formed by the heptad repeat 1 (HR1) region of gp41 are exposed [8]–[10]. These hydrophobic grooves are subsequently occupied by helices from the gp41 heptad repeat 2 (HR2) region, during the formation of an energetically stable six-helix bundle that is thought to drive the fusion of the viral and target cell membranes [9],[11],[12].

In contrast to the activating effect of cell-surface CD4 on HIV-1 entry, the soluble form of CD4 (sCD4) demonstrates opposing effects on HIV-1 infectivity at different concentrations. At high concentrations, sCD4 neutralizes most HIV-1 strains [13]; at lower sCD4 concentrations, the infectivity of some HIV-1 strains can be modestly enhanced [14]. This enhancing effect of sCD4 is more prominent in some strains of the related primate immunodeficiency viruses, HIV-2 and simian immunodeficiency virus (SIV), where sCD4 can efficiently replace cell-surface CD4 to drive infection of CD4^−^CCR5^+^ cells [15],[16].

Based on the potential of sCD4 to inhibit HIV-1 infection *in vitro*, this protein was tested for clinical efficacy in HIV-1-infected individuals; however, no effect on plasma viral loads was observed [13]. Further examination revealed that doses of sCD4 that were significantly higher than those achieved in the clinical trial were required to neutralize primary clinical isolates of HIV-1, in contrast to the relatively sensitive, laboratory-adapted strains [17].

Interest in improving the therapeutic potential of sCD4 has prompted investigation of the mechanistic basis for sCD4-induced neutralization. Competitive inhibition of envelope glycoprotein binding to the cell-surface CD4 receptor was suggested as a major mechanism of sCD4 neutralization [17],[18]. Resistance to sCD4 may thus arise by a decreased affinity of the envelope glycoprotein complex for sCD4 [13]. However, sCD4 sensitivity cannot always be predicted by measurements of affinity [17], [19]–[21]. Binding of sCD4 to the envelope glycoprotein trimer can induce detachment (shedding) of the gp120 subunit from the envelope glycoprotein trimer [17], [18], [22]–[24]. However, the sCD4 concentrations that are required to elicit shedding are significantly higher than those required to neutralize the virus [25],[26]. In addition, for some HIV-1 strains, the temperature dependence of sCD4-induced gp120 shedding and virus neutralization differs [26]. The mode of sCD4-mediated inhibition thus remains incompletely understood.

Targeting the functionally important and therefore conserved CD4-binding site on HIV-1 gp120 represents an attractive potential approach to therapy or prophylaxis. CD4-mimicking peptides and oligomeric forms of sCD4 that target this site on primary HIV-1 isolates have been developed [27]–[30]. Recently, a new class of small-molecule CD4 mimics was identified [31],[32]. These compounds, which include the prototypic compound NBD-556 and its derivatives, mimic the effects of CD4 by inducing the exposure of the coreceptor-binding site on gp120 [31],[33]. Although NBD-556 inhibits HIV-1 infection of CD4^+^CCR5^+^ cells, it can replace CD4 and thus enhance HIV-1 infection of CD4^−^CCR5^+^ cells [31]. In view of their capacity to enhance infectivity, any potential application of CD4-mimetic compounds and sCD4 derivatives, herein referred to as soluble CD4 mimics (SCMs), would benefit from a mechanistic understanding of these activating and inhibitory effects.

The study of receptor-induced conformational changes of the HIV-1 envelope glycoprotein complex is still limited by the lack of tools that enable monitoring of this dynamic multi-step process. Detection of conformational intermediates is largely based on the use of conformation-specific fusion/entry inhibitors, which are added at different times after initiation of the fusion process [34]–[38]. Although such assays have provided insights into the mechanisms of gp41-directed inhibitors, they fail to detect transitions to conformations that are inactive and do not differentiate between lack of inhibitor binding and lack of inhibition. Furthermore, because formation of the ternary complex between the envelope glycoproteins, CD4 and coreceptor constitutes a major rate-limiting step in the membrane fusion process, these approaches are limited in their capacity to define the progression of subsequent events [39]. Finally, due to the low temporal resolution of most systems, mainly steady-state conformations are detected, while intermediate states of the complex may be missed.

To overcome these limitations, we developed new systems that allow us to monitor the dynamic changes in conformation and function of the HIV-1 envelope glycoproteins, immediately after engagement of the activating molecules. Using these tools, we found that SCMs inactivate envelope glycoprotein function by an activation-triggered inhibition process, through induction of a metastable activated state.

## Materials and Methods

### Reagents and Antibodies

Four-domain sCD4 (molecular weight 50 kDa) was expressed in 293F cells after stable transfection. The protein was secreted into the cell culture medium and then purified by using the C-terminal His_6_ tag, as previously described [40]. The structure of compound JRC-II-191 (herein referred to as **191**) is reported in reference [33]. The CCR5 antagonist ‘Compound A’ [4-nitrobenzyl 1-(3-(N-methylphenylsulfonamido)-3-phenylbutyl)piperidin-4-yl(vinyl)carbamate] [41],[42] was kindly provided by Dr. Martin Springer at Merck Research Laboratories, Rahway, NJ.

The anti-gp120 monoclonal antibody 48d, which recognizes an epitope that overlaps the coreceptor-binding site, was kindly provided by Dr. James Robinson (Tulane University Medical Center) [43]. The monoclonal antibodies IgG1 b12 and 39F recognize the CD4-binding site and the V3 loop of gp120, respectively [44],[45]. The C34-Ig fusion protein consists of the Fc region of human IgG1 linked to the HR2 region of the HXBc2 envelope glycoprotein (amino acid residues 628–661, numbered according to current convention [46]). C34-Ig was produced and purified as previously described [47]. The CD4-Ig fusion protein consists of the first two N-terminal domains of the CD4 molecule and the Fc region of human IgG1. Purification was carried out as described for the C34-Ig molecule [47].

### Envelope Glycoprotein Constructs

The primary viruses UK7br and UK7br34 were isolated from autopsy brain tissue from an AIDS patient with HIV-1 associated dementia [48]. These envelope glycoproteins were expressed from the pcDNA 3.1 backbone vector. All other envelope glycoproteins described were expressed from the pSVIIIenv vector [49]. The YU2, AD8, JR-FL, 89.6 and KB9 *env* sequences from Asp 718 (Kpn I) to BamH I were substituted for the corresponding HXBc2 *env* sequences in the original pSVIIIenv vector. The ΔKS construct, which contains an HIV-1 HXBc2 *env* gene with a large deletion, was used as a negative control. The YU2(Δct) protein has a truncated cytoplasmic tail of 17 amino acids, with a stop codon introduced after Ala 710 (numbered according to current convention [46]).

The YU2-GS8 construct is a cleavage-defective form of the YU2 HIV-1 envelope glycoproteins that contains an 8-amino acid glycine-serine linker at the gp120/gp41 junction. Starting with the cytoplasmic tail-deleted YU2 envelope glycoproteins, both Arg 508 and Arg 511 near the furin cleavage site were altered to Ser to render the protein cleavage-defective. The 8-amino acid linker, Gly-Gly-Gly-Ser-Gly-Gly-Gly-Ser, was then inserted between Ser 511 (the C-terminal residue of gp120) and Ala 512 (the N-terminal residue of gp41) using the overlapping PCR method.

### Cell-Based Enzyme-Linked Immunosorbent Assay (ELISA)

A sensitive cell-based ELISA with high temporal resolution was developed to measure the binding of antibodies to HIV-1 envelope glycoprotein trimers expressed on cells. COS-1 cells were seeded in 96-well plates (2.4×10^4^ cells per well) and transfected the next day with 0.1 µg of a plasmid expressing the envelope glycoproteins and 0.01 µg of a Tat-expressing plasmid per well using the Effectene transfection reagent (Qiagen). Two days later, cells were washed twice with blocking buffer (35 mg/ml BSA, 10 mg/ml non-fat dry milk, 1.8 mM CaCl_2_, 1 mM MgCl_2_, 25 mM Tris, pH 7.5 and 140 mM NaCl). For pulse activation experiments, the COS-1 cells were incubated with sCD4 (40 µg/ml, 0.8 µM) or **191** (360 µM) suspended in blocking buffer for three minutes, washed three times with blocking buffer and incubated for different time periods until the C34-Ig or 48d antibodies were added (at 40 µg/ml or 1 µg/ml, respectively, for 30 minutes). To study the temperature dependence of HR1 groove exposure, the sCD4-pulsed cells were incubated at the requisite temperature for different lengths of time; the cells were subsequently returned to room temperature for incubation with C34-Ig. Cells were then washed four times with blocking buffer and four times with washing buffer (140 mM NaCl, 1.8 mM CaCl_2_, 1 mM MgCl_2_ and 20 mM Tris, pH 7.5). A horseradish peroxidase-conjugated antibody specific for the Fc region of human IgG was then incubated with the samples for 45 minutes at room temperature. Cells were washed 5 times with blocking buffer and 5 times with washing buffer. HRP enzyme activity was determined after the addition of 33 µl per well of a 1∶1 mix of Western Lightning oxidizing and luminol reagents (Perkin Elmer Life Sciences) supplemented with 150 mM NaCl. Light emission was measured with a Mithras LB 940 luminometer (Berthold Technologies). For experiments that measure the rate of decay of C34-Ig or 48d antibody binding, the indicated values for the amount of antibody bound were obtained by subtracting binding measured in the absence of sCD4 from the binding measured at each time point after the sCD4 pulse.

### HR1 Groove Exposure Induced by Cell-Surface CD4

293T cells cultured in 6-well plates were transfected with the pcDNA3.1-CD4 or ΔKS constructs (1 µg per 1.8×10^6^ cells) using the Effectene reagent (Qiagen). COS-1 cells cultured in 96-well plates were transfected with plasmids expressing the HIV-1 envelope glycoproteins as described above. Two days later, the 293T cells were harvested using 5 mM EDTA in PBS and added at 4°C to the monolayer of COS-1 cells (3×10^5^ 293T cells per well). Cells were subsequently centrifuged at 1,000×g for two minutes to increase contact and incubated at 4°C for 75 minutes. Samples were then washed once with blocking buffer and rapidly equilibrated to 25°C. C34-Ig was then added at different times. Detection of C34-Ig binding was performed as described above.

### Fluorescence-Activated Cell Sorting (FACS) Analysis of Ligand Binding to Cell-Surface HIV-1 Envelope Glycoproteins

Flow cytometry was used to detect the binding of C34-Ig to 293T cells that express the cytoplasmic tail-deleted (Δct) HIV-1 envelope glycoproteins [47]. Cells were cultured in 6-well plates (1.5×10^6^ cells per well) and transfected with 0.6 µg of a plasmid expressing the HIV-1 envelope glycoproteins and 0.06 µg of a Tat-expressing plasmid using the Effectene transfection reagent (Qiagen). Two days later, cells were detached using PBS supplemented with 5 mM EDTA and resuspendend in FACS buffer (PBS supplemented with 10 mg/ml BSA and 0.5 mg/ml sodium azide). Cells were then incubated with C34-Ig suspended in FACS medium, with or without sCD4, at the indicated temperature for 45 minutes. Following three washes with FACS buffer, cells were incubated at room temperature for 45 minutes in FACS buffer containing a phycoerythrin-conjugated goat anti-human IgG antibody (Sigma) and an anti-CD4 fluorescein-conjugated antibody (OKT4, eBioscience) to measure C34-Ig and sCD4 binding, respectively. Cells were subsequently washed three times with FACS buffer and analyzed with a FACScan analyzer (Becton Dickinson) using Cellquest software for data acquisition and analysis.

### Generation and Preparation of Recombinant Luciferase-Expressing Viruses

Single-round, recombinant HIV-1 viruses that express the luciferase reporter gene were generated by transfection of 293T cells using the calcium phosphate transfection method (Promega). Cells were seeded in 100-mm tissue culture dishes (approximately 4×10^6^ cells per dish) and transfected the next day with 10 µg of the HIV-1 packaging construct pCMVΔP1ΔenvpA [50], 10 µg of the firefly luciferase-expressing construct pHIvec2.luc and 2.5 µg of the plasmid expressing the HIV-1 Rev and envelope glycoproteins. Sixteen hours following transfection, the medium was changed to culture medium (Dulbecco-modified Eagle medium supplemented with 10% fetal calf serum, DMEM/FCS 10%); 12 hours later, the medium was changed to DMEM/FCS 1%. Virus-containing supernatants were collected 12 hours later, cleared of cell debris by low speed centrifugation and filtered through 0.2 µm filters. Preparations were subsequently loaded onto Float-A-Lyzer dialysis cassettes (molecular weight cutoff 100 kDa, Spectrum Labs) and dialyzed against HS buffer (140 mM NaCl, 10 mM HEPES, pH 7.3) for 24 hours at 4°C. Viral preparations were subsequently aliquoted and stored at −80°C until use.

### Magnetically Controlled Infection by sCD4-Activated HIV-1

To minimize the time interval between activation of the virus and attachment to the cells, we magnetically controlled the attachment step. As a magnetically controllable carrier, we used magnetite nanoparticles (50 nm in diameter) that are coated by a starch polymer with phosphate end groups (FluidMag PD, Chemicell). Viruses were preincubated with magnetite nanoparticles (1.2 mg/ml) for 7 minutes at room temperature, followed by incubation with sCD4 (40 µg/ml) for different time periods. Preparations were then added to a confluent monolayer of cells cultured in a 96-well plate, to which was applied a magnetic field using a Nd-Fe-B permanent magnet (OZ Biosciences). Canine thymocyte Cf2Th cells that express CCR5 or both CD4 and CCR5 were used as target cells (approximately 7×10^4^ cells per well). After a one-minute incubation at room temperature, cells were washed twice with DMEM/FCS 10% and cultured for an additional 14 hours. Cells were then detached with trypsin-EDTA and seeded again at a 1∶6 dilution. Forty-eight hours after infection, cells were lysed with passive lysis buffer (Promega) and lysates were assayed for luciferase activity. To each well was added 100 µl of luciferin buffer (15 mM MgSO_4_, 15 mM KPO_4_ [pH 7.8], 1 mM ATP, and 1 mM dithiothreitol) and 50 µl of 1 mM D-luciferin potassium salt (BD Pharmingen). Luminescence was recorded using a Berthold LB 960 microplate luminometer.

### Decay Rate of Virus Infectivity after Pulse Activation with sCD4

The decay rate of virus infectivity after activation by sCD4 was measured using magnetically-immobilized viruses. This method allows rapid pulsing and washing of the virus before addition of target cells; thus, virus infectivity shortly after activation can be measured. Virus preparations were preincubated with magnetite nanoparticles (1.2 mg/ml, suspended in HS buffer) for 7 minutes and then added to 96-well plates placed over a permanent Ne-Fe-B magnet (OZ Biosciences). After a 4-minute incubation at room temperature, wells were washed twice with culture medium. Immobilized viruses were then pulsed with 40 µg/ml (0.8 µM) sCD4 for 3 minutes, washed twice with culture medium and then incubated at room temperature for different time periods. Cf2Th-CCR5 cells suspended in culture medium (∼1×10^5^ cells) were then added to the wells and centrifuged at 1,000×g for 2 minutes to expedite contact between cells and viruses. Luciferase activity was measured two days later.

### Decay Rate of Virus Infectivity after Pulse Activation with Compound 191

The affinity of NBD-556 analogues, including **191**, for the HIV-1 gp120 envelope glycoprotein is significantly lower than that of sCD4 [31],[33]. Measured by isothermal titration calorimetry, the K_d_ of **191** binding to the YU2 gp120 envelope glycoprotein is 760 nM, compared with a K_d_ of approximately 2 nM for the sCD4-gp120 interaction. At achievable concentrations, **191** induced significantly lower levels of HIV-1 activation than sCD4 (see Results section); levels of infectivity were consequently too low to be monitored for decay. We therefore introduced a number of modifications to the method described above for measuring infectivity decay. Briefly, viruses were incubated with 180 µM **191** at 37°C for 10 minutes. Activation was then halted by a 20-fold dilution of the virus-compound mix in HS buffer equilibrated to 26°C. After dilution, no additional activation of HIV-1 infection was induced by **191** during the time frame of the experiment (data not shown). Samples were then incubated for different time periods, associated with magnetite nanoparticles and magnetically adsorbed to confluent cultures of Cf2Th-CCR5 cells, as described above.

### Infectivity of Cell-Bound Virus Activated by sCD4

Cf2Th cells cultured in 6-well plates (6×10^5^ cells per well) were transfected with different amounts of the pcDNA3.1-CCR5 plasmid, which expresses human CCR5, using the Effectene transfection reagent. Two days later, cells were detached using 5 mM EDTA in PBS and re-seeded in 96-well plates (7×10^5^ cells per well). Viruses were then magnetically adsorbed to the transfected cells in the presence of 20 µg/ml (400 nM) sCD4 in the culture medium and infectivity was measured two days later.

## Results

### Rescue of the Infectivity of sCD4-Treated HIV-1 by Rapid Attachment to Cells

**Figure 1:**
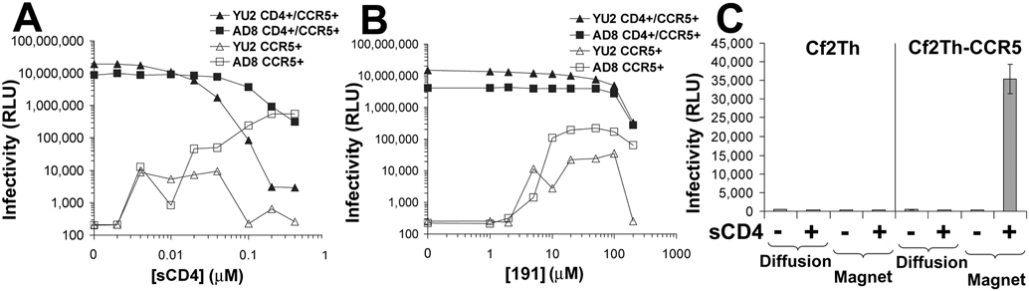
Inhibition and activation of infection by sCD4 and the CD4-mimetic compound 191. Recombinant HIV-1(YU2) or HIV-1(AD8) strains were incubated with CD4^−^CCR5^+^ or CD4^+^CCR5^+^ Cf2Th cells for 48 hours in the presence of sCD4 (A) or 191 (B). Infectivity was determined by measurement of luciferase activity. (C) HIV-1(YU2) was incubated for three minutes in the presence or absence of sCD4 (40 µg/ml, 0.8 µM) and then adsorbed by diffusion or magnetically to cultures of the indicated cell type. Measured luciferase activity is presented as mean relative light units (RLU)±standard error of the mean (s.e.m.) of three replicate samples.

Soluble CD4 mimics (SCMs) can exert varied effects on HIV-1 infection of CD4^+^ and CD4^−^ target cells. Although soluble CD4 (sCD4) typically inhibits HIV-1 infection of CD4^+^CCR5^+^ cells, enhancement of HIV-1 infection of CCR5^+^ cells lacking CD4 is sometimes seen after sCD4 treatment [14]. Sensitivity to these effects varies among different HIV-1 strains [49]. We examined the sensitivity to sCD4 of recombinant HIV-1 viruses (designated HIV-1(AD8) and HIV-1(YU2), respectively) pseudotyped with the envelope glycoproteins of the primary HIV-1 isolates AD8 and YU2, which have similar binding affinities for sCD4 [51],[52]. In CD4^+^CCR5^+^ target cells, sCD4 inhibited the infectivity of HIV-1(AD8) and HIV-1(YU2) with IC_50_ values of 80 nM (4 µg/ml) and 12 nM (0.6 µg/ml), respectively (Figure 1A). In CD4^−^CCR5^+^ cells, sCD4 activated infection of HIV-1(AD8) much more efficiently than that of HIV-1(YU2).

A similar pattern of HIV-1 activation and inactivation was observed for the NBD-556 analogue, JRC-II-191, herein referred to as **191**. The affinity of **191** for the HIV-1_YU2_ gp120 envelope glycoprotein (K_d_ = 760 nM) is significantly lower than that of sCD4 (K_d_ = ∼2 nM). Nevertheless, at proportionately higher concentrations, **191** resembled sCD4, inhibiting HIV-1 infection of CD4^+^CCR5^+^ cells and enhancing infection of CD4^−^CCR5^+^ cells (Figure 1B). For both sCD4 and **191**, the enhancement of infection of cells lacking CD4 became progressively less efficient at higher concentrations; in some cases, decreases in viral infectivity were associated with escalating doses. These characteristics of HIV-1 envelope glycoproteins differ from those observed with several SIV envelope glycoproteins, which demonstrate increasingly efficient infection of CD4^−^CCR5^+^ cells with increasing sCD4 concentrations (data not shown, and ref. [15]). These observations suggested the existence of a concentration-dependent, virus-inhibitory process superimposed on the HIV-1 activation process.

Because virus attachment to cells in culture progresses in a slow, diffusion-dependent manner [53],[54], significant time intervals can elapse between virus binding by SCMs and the association between virus and cell. Thus, the diminished capacity of the SCMs to enhance HIV-1 infection of CD4^−^CCR5^+^ cells at higher concentrations might reflect the involvement of time-dependent as well as concentration-dependent processes that abrogate infectivity after activation occurs. To test this possibility, we examined the effect of neutralizing concentrations of sCD4 on HIV-1(YU2) infection when the time interval between sCD4-virus binding and virus-cell attachment was reduced. For this purpose, we applied a system that magnetically controls virus motion to eliminate viral dependence on passive diffusion for cell attachment; this system allows near-complete transfer of the virus inoculum to the cell-bound state within one minute [53],[55]. When HIV-1(YU2) was briefly incubated with a high concentration of sCD4 and then added to CD4^−^CCR5^+^ cells, infection occurred only when the virus was magnetically adsorbed to the cells (Figure 1C). These results suggested the possibility of a time-dependent process that decreases HIV-1 infectivity after activation by sCD4, and prompted us to measure the stability of the sCD4-activated intermediate form of the HIV-1 envelope glycoproteins.

### Conformational Stability of the CD4-Activated Intermediate

Engagement of CD4 induces a major structural rearrangement of the HIV-1 envelope glycoproteins [2]. Two functionally important regions of the HIV-1 envelope glycoproteins are formed and exposed as a result of CD4 binding: i) the coreceptor-binding site on the gp120 envelope glycoprotein [3]–[7],[56]; and ii) the trimeric coiled coil composed of the N-terminal heptad repeat (HR1) regions of the three gp41 subunits [8]–[10],[47]. To investigate the conformational stability of the sCD4-activated envelope glycoprotein intermediate, we monitored changes over time in the exposure of the coreceptor-binding site on gp120 and the HR1 coiled coil on gp41 after pulse activation by sCD4.

**Figure 2:**
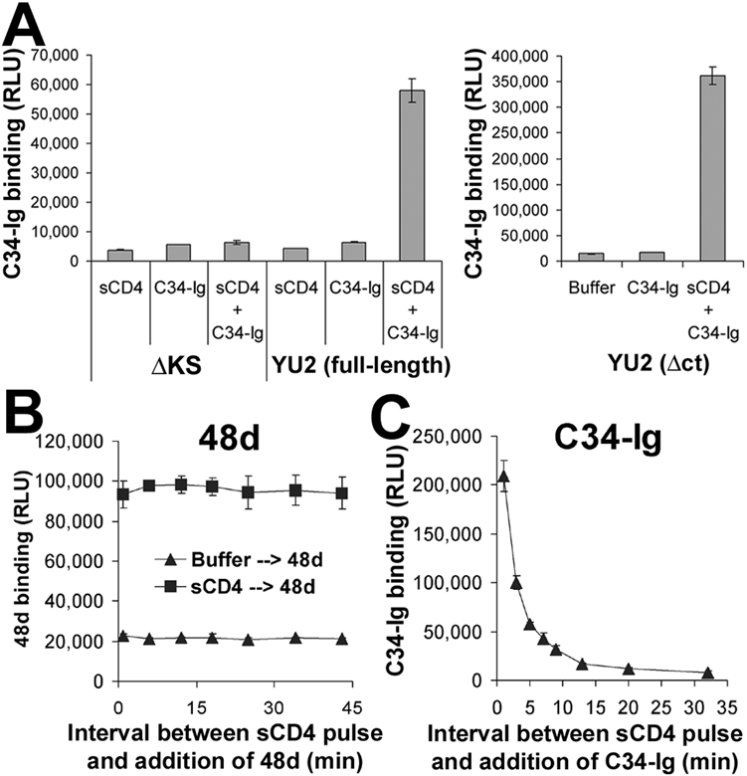
Soluble CD4-induced changes in the gp120 coreceptor–binding region and gp41 HR1 region. (A) A cell-based ELISA was used to measure the binding of C34-Ig, which detects the HR1 gp41 region [38],[47], to the trimeric HIV-1 envelope glycoproteins. COS-1 cells were transfected with the negative control ΔKS plasmid or plasmids expressing the full-length YU2 envelope glycoproteins (left) or the cytoplasmic tail-deleted (Δct) YU2 envelope glycoproteins (right). Two days later, cells were incubated with C34-Ig (20 µg/ml), in the presence or absence of sCD4 (20 µg/ml). C34-Ig binding was measured using a secondary horseradish peroxidase-conjugated antibody. (B,C) Change over time in the exposure of CD4-induced epitopes. Cells that express the YU2(Δct) envelope glycoproteins were pulsed for three minutes with sCD4 (40 µg/ml). Cells were then washed three times and incubated for different time periods at 37°C. The monoclonal antibody 48d (B) or C34-Ig (C) was then added. The bound 48d or C34-Ig molecules were detected using a horseradish peroxidase-conjugated anti-human IgG Fc antibody, as described in Materials and Methods. Values represent the mean RLU (±s.e.m.) of two replicate samples.

To measure the progression of conformational changes that immediately follow HIV-1 envelope glycoprotein activation, we developed a novel enzyme-linked immunosorbent assay (ELISA)-based system that utilizes live cells as a platform for expression of membrane-bound trimeric envelope glycoprotein complexes The system allowed sensitive detection of ligand binding to full-length envelope glycoproteins at high temporal resolution. COS-1 cells that express the HIV-1_YU2_ envelope glycoproteins were pulse-activated with sCD4 at 37°C for 3 minutes and incubated at 37°C for different time periods. The following probes were then added: i) monoclonal antibody 48d, which binds to a gp120 epitope that overlaps the coreceptor-binding site [43]; or ii) the C34-Ig protein, which is composed of the C34 peptide corresponding to the gp41 HR2 region linked to the Fc constant region of human IgG1 [47]. C34-Ig binds to the hydrophobic groove formed at the interface of the HR1 coiled-coil helices [10],[47]. The sCD4 pulse significantly enhanced the binding of the C34-Ig protein and the 48d antibody to the HIV-1 envelope glycoproteins (Figure 2A and 2B). The time interval between the sCD4 pulse and addition of the 48d antibody did not affect the level of 48d binding observed (Figure 2B). Thus, after the sCD4-induced conformational change, the epitope of the 48d antibody remains stably exposed over the time period examined.

**Figure 3:**
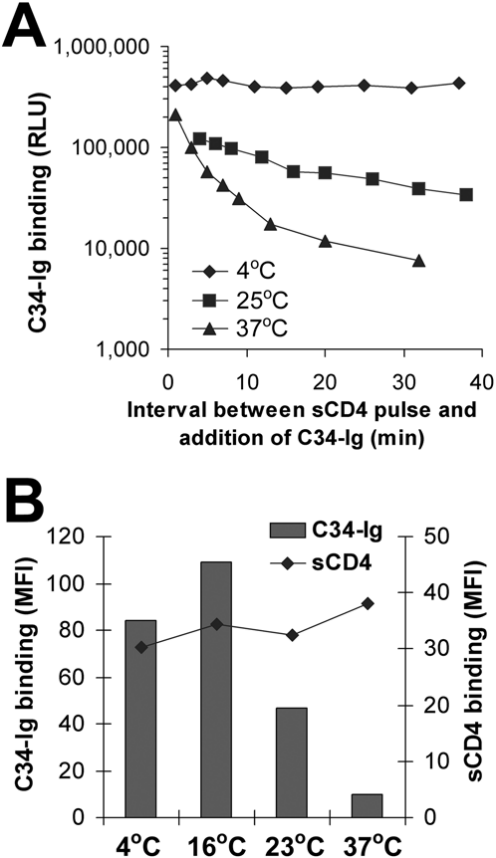
Temperature dependence of the decay of HR1 groove exposure. (A) Decay of HR1 groove exposure at different temperatures after pulse activation with sCD4 (40 µg/ml; 0.8 µM) was measured by a cell-based ELISA, as described in Materials and Methods. The indicated values for C34-Ig binding were obtained by subtracting the values of C34-Ig binding in the absence of sCD4 from the binding measured at each time point after the sCD4 pulse. Mean background levels of C34-Ig binding (in the absence of sCD4) were 10316, 21413, and 19028 RLU for the experiments performed at 4°C, 25°C, and 37°C, respectively. (B) Binding of C34-Ig and sCD4 to the HIV-1 envelope glycoproteins was measured at different temperatures. Cells that express the YU2(Δct) envelope glycoproteins were incubated simultaneously with both C34-Ig (20 µg/ml) and sCD4 (20 µg/ml; 0.4 µM) at the indicated temperature. Binding of sCD4 and C34-Ig was detected by FACS analysis using a fluorescein-conjugated anti-CD4 antibody and a phycoerythrin-conjugated goat anti-human IgG antibody, respectively (see Materials and Methods). The mean fluorescence intensity (MFI) of the cells in both channels is shown.

In contrast to the above results, the exposure of the C34-Ig binding site after the sCD4 pulse was transient at 37°C (Figure 2C). Stability of the HR1-exposed state was highly dependent on the temperature of incubation (Figure 3A). Although sCD4 efficiently bound to the envelope glycoprotein complex and induced the formation/exposure of the HR1 groove at 4, 25 and 37°C, this state was stable only at lower temperatures (Figure 3B). The loss of exposure of the HR1 groove was not reversible by repeated pulsing of the envelope glycoprotein complexes with saturating concentrations of sCD4 (data not shown). At the different temperatures examined, in the absence of sCD4, C34-Ig binding was similar to that measured for the negative control cells transfected with the ΔKS plasmid. No correlation was observed between the temperature of incubation and the binding of C34-Ig in the absence of sCD4.

Given the constant level of 48d antibody binding to the sCD4-pulsed envelope glycoprotein-expressing cells, the observed time-dependent decrease in C34-Ig binding cannot be attributed to changes in cell-surface levels of the envelope glycoproteins, as might occur due to gp120 shedding [17]. Moreover, we observed that sCD4 binding to the cells remained constant over the time period examined. We performed a kinetic experiment to measure the decay of HR1 exposure using FACS analysis. The binding of sCD4 and C34-Ig to the envelope glycoprotein-expressing cells was measured simultaneously at different time points after the sCD4 pulse, using phycoerythrin- and fluorescein-conjugated secondary antibodies. We observed a gradually decreasing capacity of C34-Ig to bind despite constant levels of bound sCD4 (Figure S1).

In summary, after engagement of sCD4, the HIV-1 envelope glycoprotein complex undergoes conformational changes that result in formation/exposure of the gp120 coreceptor-binding site and the gp41 HR1 groove. However, despite continued engagement of sCD4 and stable exposure of the 48d epitope on gp120, the induced envelope glycoprotein intermediate undergoes a spontaneous temperature-dependent and apparently irreversible change of conformation.

### Decay of Exposure of the HR1 Groove on Envelope Glycoproteins from Different HIV-1 Strains

**Figure 4:**
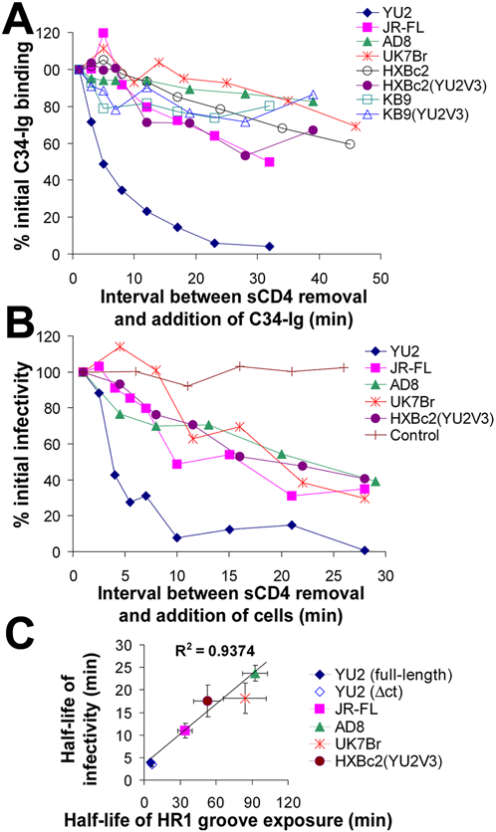
Relationship between infectivity decay and loss of HR1 groove exposure. (A) Decay of HR1 groove exposure of different HIV-1 envelope glycoproteins was studied at 25°C after pulse activation with sCD4. COS-1 cells expressing the indicated HIV-1 envelope glycoproteins were pulsed with sCD4 (40 µg/ml; 0.8 µM) for 3 minutes, followed by assessment of HR1 groove exposure using the cell-based ELISA method, as described in the Figure 2 legend. (B) Recombinant HIV-1 virions carrying the indicated envelope glycoproteins were pulsed with sCD4 (40 µg/ml; 0.8 µM) for 3 minutes and incubated at 25°C. After the indicated times, CD4^−^CCR5^+^ Cf2Th cells were added to the viruses. For the sample marked as control, HIV-1(YU2) was pulsed with buffer and then CD4^+^CCR5^+^ cells were added. Two days later, virus infectivity was assessed by measuring luciferase activity in the target cells. (C) The relationship between the decay rate of HR1 groove exposure and the decay rate of infectivity, both measured at 25–27°C after pulse activation with sCD4, is shown for the panel of HIV-1 envelope glycoproteins. Half-lives were determined by fitting a model function to the data using nonlinear regression. Values represent half-lives (±s.e.m. for both variables) derived from two to four separate experiments for each envelope glycoprotein variant.

The decay profile of sCD4-induced HR1 groove exposure was determined for a panel of envelope glycoproteins from primary and laboratory-adapted HIV-1 strains. Measurements were conducted at room temperature (24–26°C) to slow the rate of decay, allowing greater accuracy. Interestingly, a wide range of stabilities of the HR1 groove-exposed state was observed in the different HIV-1 envelope glycoproteins (Figure 4A). For example, the YU2 and AD8 envelope glycoproteins exhibited half-lives of 5 and 100 minutes, respectively. The different decay rates of HR1 groove exposure could not be explained by differences in the affinity of the trimeric envelope glycoprotein complexes for sCD4 (Figure S2A), or in kinetic differences in CD4-Ig binding to these complexes (Figure S2B). We also found that the deletion of the gp41 cytoplasmic tail had no effect on the stability of the CD4-activated intermediate (Figure S2C), even though the cell-surface level of envelope glycoprotein expression was increased, as expected [57].

Envelope glycoproteins from HIV-1_89.6_ and HIV-1_KB9_ exhibited highly stable sCD4-induced intermediates that did not appreciably decay at room temperature for up to 1 hour after the sCD4 pulse. For these envelope glycoproteins, the half-lives of the HR1-exposed state measured at 37°C were 16 and 17 minutes, respectively (Figure S2D).

### Correlation between Stability of the HR1 Groove and Functional Stability of the HIV-1 Envelope Glycoproteins after sCD4 Exposure

Does the sCD4-induced conformational instability in the HIV-1 envelope glycoproteins have consequences for virus infectivity? To examine this question, we devised a strategy to measure changes over time in the capacity of sCD4-activated virus to infect CD4^−^CCR5^+^ cells. First, recombinant, luciferase-expressing viruses were associated with magnetite nanoparticles and immobilized on tissue-culture plates by constant application of a magnetic field. Next, the immobilized viruses were pulsed with sCD4 for 3 minutes and incubated at 25°C for different time periods. Then, CD4^−^CCR5^+^ cells were added and immediately pelleted onto the immobilized viruses. Infectivity was assessed by measuring luciferase activity in the cells 48 hours later. The levels of initial infectivity for viruses pseudotyped with the different HIV-1 envelope glycoproteins were significantly increased by incubation with sCD4 (data not shown). However, infectivity subsequently declined at different rates for viruses with different envelope glycoproteins (Figure 4B). Similar to the stability of HR1 groove exposure, the rate of decay of infectivity was highly dependent upon temperature (Figure S3). At room temperature, the half-life of infectivity exhibited by the sCD4-treated viruses pseudotyped with different HIV-1 envelope glycoproteins correlated well with the half-life of the HR1 groove-exposed state following incubation with sCD4 (R^2^ = 0.9374, Figure 4C).

Assuming that the linear regression model generated using the data obtained at 25°C applies to the situation at 37°C, we first estimated the half-lives of HIV-1 infectivity by fitting the half-lives of HR1 groove exposure measured at 37°C to the model. For the most stable envelope glycoproteins (derived from HIV-1_89.6_ and HIV-1_KB9_), we estimated a maximal half-life of infectivity of 5–7 minutes at 37°C. In accordance with the predicted values, the measured half-lives of infectivity at 37°C for viruses containing the KB9 and 89.6 envelope glycoproteins were 7 and 8 minutes, respectively, after the sCD4 pulse (Figure S2E).

We compared the infectious half-lives of viruses with different HIV-1 envelope glycoproteins following incubation with sCD4 (using CD4^−^CCR5^+^ cells, as described above) and without sCD4 treatment (using CD4^+^CCR5^+^ cells). Incubation of the viruses at 37°C for increasing time periods in the absence of sCD4, followed by infection of CD4^+^CCR5^+^ cells, revealed a gradual reduction of infectivity, with half-lives ranging from 6.9 to 12.1 hours (Figure S4). However, no correlation between the functional stability of the native and sCD4-bound states of each of the envelope glycoproteins was observed.

We conclude that after engagement of sCD4, HIV-1 undergoes a transient phase of activation, during which attachment to cells that express CCR5 will allow entry to occur. However, this activated state is short-lived and is followed by apparently irreversible structural rearrangements and loss of infectivity. Thus, at any given time point after exposure to sCD4, HIV-1 infectivity represents the combined result of an activation process and progression through the activated intermediate state to inactivation (Figure S5).

### Metastable Activation Induced by a Small-Molecule CD4 Mimic

**Figure 5:**
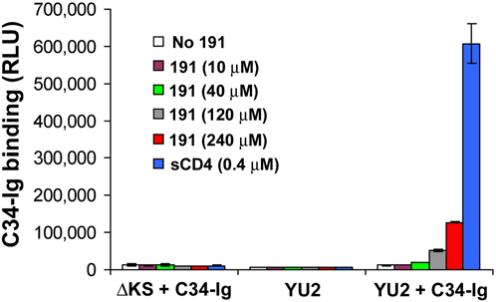
Effect of compound 191 on exposure of the gp41 HR1 groove. COS-1 cells transfected with a plasmid expressing the YU2(Δct) envelope glycoproteins or with the control ΔKS plasmid, which does not express an envelope glycoprotein on the cell surface, were incubated with the indicated molecules in the presence or absence of C34-Ig (20 µg/ml). The data represent the means (±s.e.m.) of two replicate samples.

**Figure 6:**
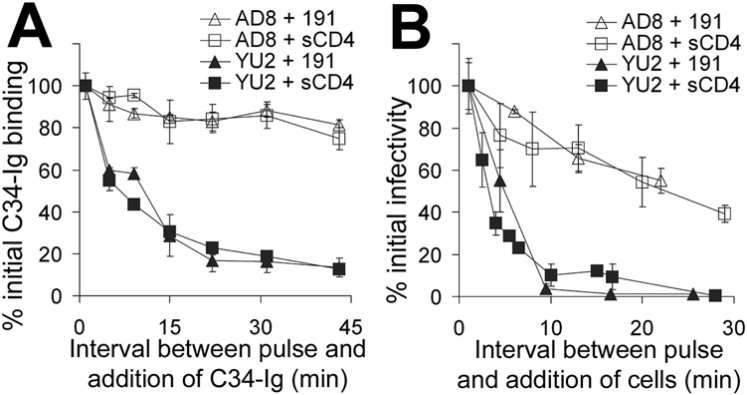
Longevity of the HIV-1 envelope glycoprotein intermediate at room temperature after activation by sCD4 or 191. (A) The decay of HR1 groove exposure for cell-surface–expressed YU2 and AD8 envelope glycoproteins is compared after pulse activation with 191 (360 µM) or sCD4 (40 µg/ml, 0.8 µM). (B) The decay of the ability to infect CD4^−^CCR5^+^ Cf2Th cells after pulse activation with 191 or sCD4 is compared.

CD4-mimetic compounds exemplified by NBD-556 and **191** induce conformational changes in the HIV-1 envelope glycoproteins similar to those induced by sCD4 [31],[33]. These changes include formation/exposure of both the coreceptor-binding site on gp120 and the HR1 groove on gp41 (Figure 5). The similarity between the effects of **191** and sCD4 on HIV-1 infection of CD4^+^ and CD4^−^ cells (Figure 1) suggested that **191** may also induce transiently activated intermediate states in the HIV-1 envelope glycoproteins. To examine this possibility, we compared the decay profiles associated with sCD4 and **191**. We examined the YU2 and AD8 envelope glycoproteins, which exhibit dramatic differences in the decay rate of the sCD4-induced intermediate. For each of these envelope glycoproteins, sCD4 and **191** induced identical decay profiles, both for HR1 groove exposure and for infectivity (Figure 6). These results suggest that sCD4 and **191** induce a similar, transient intermediate state in the HIV-1 envelope glycoproteins. Moreover, the differences in decay rates between the AD8 and YU2 envelope glycoproteins were evident for both sCD4 and **191**, suggesting that the stability of the induced state is determined by intrinsic properties of the envelope glycoproteins rather than by the activating agent.

### A More Stable Envelope Glycoprotein Intermediate Induced by Cell-Surface CD4

**Figure 7:**
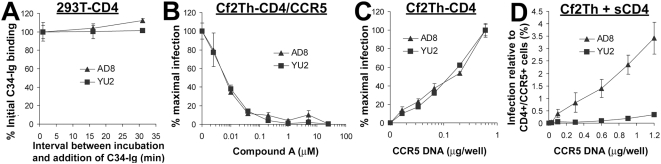
HIV-1 activation by native CD4 and sCD4. (A) The stability of HR1 groove exposure was measured after activation of the YU2 or AD8 envelope glycoproteins by cell-surface CD4. Background was defined as C34-Ig binding to cells transfected with the YU2-GS8 construct, which engages sCD4 with an affinity similar to that of the wild-type YU2 envelope glycoproteins but does not expose the HR1 groove (see Figure S6). The background was subtracted from all measurements, which are presented as percent (±s.e.m.) of C34-Ig binding measured at the initial time point. (B) The effect of the CCR5 antagonist “Compound A” [41],[42] on infectivity of recombinant HIV-1 pseudotyped with the indicated envelope glycoproteins was investigated. Cf2Th-CD4/CCR5 cells were infected with HIV-1(AD8) or HIV-1(YU2) in the presence of increasing concentrations of Compound A. Data are presented as the percentage (±s.e.m.) of infection measured in the absence of the compound. (C) The effect of CCR5 expression on the infection of CD4-expressing cells by HIV-1(AD8) or HIV-1(YU2) was examined. Cf2Th-CD4 cells were transfected with different amounts of a plasmid that expresses human CCR5. Two days later, transfected cells were incubated with HIV-1(AD8) or HIV-1(YU2). Infectivity is expressed as the percentage (±s.e.m.) of infectivity measured for cells transfected with the highest amount of the CCR5-expressing plasmid. (D) The graph shows infection by cell-bound virus of CD4^−^ Cf2Th cells transfected with different amounts of the CCR5-expressing plasmid in the presence of 20 µg/ml sCD4. Infectivity is expressed as the percentage (±s.e.m.) of infection measured in cells transfected with plasmids expressing CD4 and CCR5 (0.6 and 0.9 µg, respectively, of each plasmid per well).

To examine the longevity of the activated intermediate induced during the normal HIV-1 entry process, we examined the stability of HR1 groove exposure after the binding of the envelope glycoproteins to cell-surface CD4. For this purpose, 293T cells that express CD4 (but not CCR5) were used to activate the HIV-1 envelope glycoproteins expressed on COS-1 cells. Pilot experiments demonstrated that the observed increases in C34-Ig binding to the envelope glycoproteins-expressing cells depended on the presence of CD4 on the activating cell, and were not observed for a mutant envelope glycoprotein (YU2-GS8), which binds CD4 efficiently but does not expose the HR1 groove in response (see Figure S6 and Materials and Methods). Relative to the effects of treatment with sCD4 or **191**, the HR1 groove exposure consequent to activation by cell-surface CD4 was surprisingly long-lived (Figure 7A). The AD8 and YU2 envelope glycoproteins, which exhibited significant differences in the half-lives of HR1 groove exposure after activation by sCD4, both formed stable intermediates following activation by cell-surface CD4.

During HIV-1 entry, CCR5 binding to the CD4-induced envelope glycoprotein intermediate promotes progression along the path to virus entry [58]. Differences in the longevity of the CD4-induced state would be predicted to manifest themselves as an altered dependency on the density of target cell CCR5. To test this, the CCR5 available on CD4-expressing cells for interaction with the HIV-1 envelope glycoproteins was varied in two ways: 1) the addition of increasing amounts of Compound A, a CCR5 inhibitory compound [41],[42],[59], to cells expressing both CD4 and CCR5; and 2) transfection of different amounts of a CCR5-expressing plasmid into cells expressing CD4. The latter method is based on the direct relationship between the amount of CCR5 DNA transfected and the cell-surface density of the expressed CCR5 molecule (Figure S7). The cells were then exposed to HIV-1(AD8) or HIV-1(YU2) and the efficiency of infection measured. HIV-1(AD8) and HIV-1(YU2) behaved indistinguishably in response to variations in the CCR5 available on target cells expressing CD4 (Figure 7B and 7C). In contrast, when sCD4 was used to activate infection of Cf2Th cells expressing different levels of CCR5, entry by HIV-1(AD8) was significantly better than that of HIV-1(YU2) (Figure 7D). These results are consistent with the existence of a relatively long-lived envelope glycoprotein intermediate being induced by CD4 expressed on the target membrane.

## Discussion

Clinical testing of the inhibitory efficiency of sCD4 revealed no reduction of viral loads in HIV-1-infected individuals [13]. Failure of treatment was attributed to the resistance to sCD4-mediated inhibition of primary HIV-1 isolates, relative to laboratory-adapted isolates [13]. However, lower sCD4-binding affinity could not be consistently correlated with resistance of HIV-1 isolates to inhibition [17],[19]. Furthermore, in vitro observations of sCD4-mediated enhancement of infection [14] remained largely unexplained. More recently, small-molecular-weight compounds have been developed that target the highly conserved CD4-binding site of the gp120 envelope glycoprotein [31],[32]. NBD-556 and its derivatives bind to the gp120 envelope glycoprotein and induce structural changes similar to those promoted by sCD4. Although currently NBD-556 and analogues are only interesting tools to study the entry process, with improvement, they could prove useful therapeutically or prophylactically. However, in view of their potential to enhance HIV-1 infection, an understanding of the mechanisms underlying the effects of SCMs is essential for the safe application of these inhibitors.

In this study, we apply a new approach to define the dynamics of conformational changes that occur in the trimeric membrane-bound form of the HIV-1 envelope glycoproteins. The binding of conformation-specific probes was directly measured at high temporal resolution, allowing us to monitor transiently exposed structures and thus to define the longevity of specific activated intermediates. The lack of dependence on indirect methods, such as the use of conformation-specific inhibitors, and the capacity to detect conformational intermediates permitted identification of an SCM-induced transition to the inactive state. In contrast to previous observations based on the interactions of envelope glycoprotein- and receptor-expressing cells [26],[60], we detected no lag in the formation/exposure of either the gp120 coreceptor-binding site or the gp41 HR1 groove, two key signatures of the SCM-activated state. Even at low temperatures, both sites were immediately exposed after binding of the SCMs to gp120. This is consistent with a model in which conformational changes in the envelope glycoproteins associated with SCM binding are tightly coupled, or perhaps coincide, with the structural transitions that shape and expose the coreceptor-binding site and the HR1 coiled coil.

Except for its lower affinity for the envelope glycoproteins, the NBD-556 analogue **191** [31],[33] exerted effects that were remarkably similar to those exerted by sCD4. Thus, the properties of the SCM-induced intermediate defined in this study are intrinsic to the envelope glycoproteins of the particular HIV-1 strain rather than being dependent on the individual SCMs.

During HIV-1 infection of CD4^+^ cells, SCMs compete for cell-associated CD4. Competition for the cell-surface CD4 receptor was previously suggested as a major mechanism of HIV-1 inhibition by sCD4 [17],[18]. In this work, we show that SCMs can efficiently substitute for cell-surface CD4. In this context, some inhibition of infection may result from the difference in the efficiency with which membrane-anchored CD4 and SCMs promote HIV-1 entry. Importantly, we demonstrate that SCMs inhibit HIV-1 infection by a novel mechanism that is initiated by activation of the viral envelope glycoproteins. Similar to the envelope glycoprotein intermediate that results from binding cell-surface CD4, the SCM-induced activated state is characterized by the exposure of the coreceptor-binding site on gp120 and the HR1 groove on gp41. Moreover, the SCM-activated envelope glycoproteins are able to mediate virus entry into CCR5^+^ cells lacking CD4. However, in contrast to the stable activation induced by cell-surface CD4, the SCM-activated intermediate decays rapidly, in a manner dependent upon temperature and envelope glycoprotein strain. In the process, both exposure of the HR1 groove and membrane-fusing potential are irreversibly lost.

The SCM-induced exposure of the hydrophobic HR1 groove may be energetically unfavorable in the context of free virus, favoring a transition to a more stable but nonfunctional state. Whether this nonfunctional state involves a prematurely triggered six-helix bundle in gp41 or another, possibly off-pathway, conformation requires further investigation. Arguing against the former possibility is the expectation that six-helix bundle formation in gp41 would be incompatible with maintaining the non-covalent association with gp120. In fact, even for the most unstable envelope glycoprotein intermediate studied, no evidence of gp120 shedding after SCM treatment was observed. Despite rapid loss of function, the SCM-inactivated envelope glycoproteins retained gp120, which remained associated with the SCM and stably attached to the expressing cell surface. This finding is consistent with previous studies of dose response, temperature dependence, timing and HIV-1 strain specificity that show a lack of correlation between the HIV-1-inhibitory activity of sCD4 and the degree of gp120 shedding induced [14],[17],[19],[21],[25].

By contrast, the kinetics of the decay of SCM-induced HR1 groove exposure and the decay of HIV-1 infectivity correlate, and the ranges of SCM concentrations required for induction of the labile envelope glycoprotein intermediate and for virus neutralization overlap. These observations support the important contribution that induction of the metastable activated intermediate makes to SCM-mediated inhibition of virus infectivity. Achieving inhibition by initiating the targeted process is unusual, but two properties of the HIV-1 envelope glycoproteins render them susceptible to this strategy of inhibition: 1) initial folding and assembly into a high-potential-energy form that is prone to transform into energetically more favorable states; and 2) triggering of these conformational transitions by receptor binding, allowing a receptor mimic to take advantage of the built-in propensity of the viral envelope glycoproteins to engage the receptor and undergo global conformational changes.

The efficiency of an activation-based mechanism of inhibition is determined by the change over time in the distribution of the HIV-1 envelope glycoproteins among three states: 1) unliganded/non-activated; 2) bound/activated; and 3) post-activation decayed. This distribution is influenced by the following factors: 1) rate of SCM engagement; 2) the time interval between SCM-induced activation and progression to a step of infection that is unaffected by the decay process; and 3) the intrinsic stability of the SCM-induced activated intermediate. Forces that determine the distribution between these three states dictate the balance between enhancement and inhibition of infection, as detailed below.

Infection by cell-free virus is rate-limited by the slow diffusion-dependent attachment of the virus to the cell surface [53],[54]. Activation of the diffusing virus by a soluble molecule is thus characterized by an extended lag period between activation and the next step of the infection sequence. Half-maximal productive adsorption of diffusing HIV-1 virions to a cell monolayer (i.e., attachment that culminates in an infection event) occurs after approximately 5 hours at 37°C [55]. By contrast, the half-life of infectivity of the most stable activated intermediate examined in this study at 37°C was less than 8 minutes. As the longevity of the SCM-activated intermediate is significantly shorter than the duration of the attachment step, SCM-mediated inhibition of cell-free virus is primarily determined by the rate of activation (i.e. by both the on-rate and effective concentration of the compound).

Activating effects on HIV-1 infection predominate at low concentrations of SCMs. The dependence of virus activation and inactivation on SCM concentration was particularly evident in the biphasic dose-effect curves associated with HIV-1(YU2) infection of CD4^−^ CCR5^+^ target cells (Figure 1A and 1B and Figure S5). The stoichiometry of SCM binding to the HIV-1 envelope glycoprotein trimers may influence the propensity of the activated intermediate(s) to proceed along entry or post-activation decay pathways. For example, the binding of two SCMs to the envelope glycoprotein trimer may be more efficient in promoting inactivation/decay than the binding of a single SCM. Such a model would explain the exceptionally long-lived sCD4 activation of SIV [15],[61], the envelope glycoproteins of which have been reported to bind only one CD4 molecule per trimer [62],[63].

Retrovirus transmission is significantly more efficient when virus is transferred through direct physical interaction between cells (cell-cell transmission) than by diffusion of virions in cell-free transmission [64]. During cell-cell transmission, where HIV-1 virions emerging from the infected cell rapidly achieve proximity to the target cell [65],[66], SCMs are more likely to enhance infection than in the case of cell-free infection. Indeed, HIV-1 that was pre-bound to the surface of CD4^−^CCR5^+^ cells, perhaps mimicking the conditions of cell-cell transmission, infected the cells efficiently after incubation with SCM concentrations that were highly neutralizing for cell-free virus. In cell-cell transmission, the time interval between SCM-induced activation and the next step committed to the infection pathway exerts a dominant influence on the efficiency of HIV-1 inhibition by SCMs. Several observations suggest that the engagement of the coreceptor plays a major role in moving the SCM-activated HIV-1 envelope glycoproteins along the entry pathway. First, the sCD4-mediated enhancement of HIV-1 infection of CD4^−^ cells is highly dependent on the level of CCR5 expression (Figure 7D). Second, a study of activation of infection of CD4^−^CCR5^+^ cells by NBD-556 indicated a contribution of CCR5-binding affinity to susceptibility to enhancement [33]. Finally, although SCMs allowed CCR5-using viruses to infect CD4^−^CCR5^+^ cells, SCMs did not stimulate the infection of CD4^−^CXCR4^+^ cells by CXCR4-using viruses. The lack of enhancement of CXCR4-using viruses was not due to greater lability of the activated state. The half-life of the sCD4-induced, HR1-groove-exposed state on the CXCR4-tropic HXBc2 envelope glycoproteins was 55 minutes at 26°C, and was significantly longer on the dual-tropic KB9 envelope glycoproteins. Despite this, neither of these envelope glycoproteins supported infection of CD4^−^CXCR4^+^ cells after incubation with sCD4 or **191** [ref. 33 and data not shown]. However, **191** did allow viruses with the KB9 envelope glycoproteins to infect CD4^−^CCR5^+^ cells. The apparent inability of SCM-activated viruses to utilize CXCR4 for entry may be a consequence of the significantly lower affinity of CXCR4 for the HIV-1 envelope glycoproteins, relative to that of CCR5 [67]. Together, these observations support the importance of efficient coreceptor binding to the susceptibility of HIV-1 to SCM-induced activation of infection of CD4^−^ cells. Further work will be required to assess whether this type of enhancement can occur on primary CD4^−^CCR5^+^ cells following SCM treatment.

The mechanism of inhibition elucidated here suggests that SCMs will be most effective in settings, such as sexual transmission, in which HIV-1 is fully dependent on diffusion for successful infection. Efforts to develop small-molecule SCMs with improved rates of engaging the HIV-1 envelope glycoproteins are therefore warranted.

Binding of the HIV-1 envelope glycoproteins to native, membrane-anchored CD4 resulted in the stable exposure of the HR1 groove on gp41, in contrast to the labile structure induced by SCMs. Although additional studies will be needed to address the basis of this difference, our data rule out the contribution of CCR5 or CXCR4 on the target cell. The stable nature of the activated HIV-1 envelope glycoprotein intermediate induced by cell surface CD4 facilitates engagement of additional CD4 molecules and the coreceptor, and creates a “window of opportunity” for inhibition of downstream events in virus entry.

## Supporting Information

Figure S1 Decay of HR1 groove exposure measured by FACS. Cells that express the YU2(Δct) envelope glycoproteins were incubated at room temperature with sCD4 (40 µg/ml; 0.8 µM) for 20 min, washed, and further incubated at room temperature for the indicated time periods. Then the C34-Ig protein (40 µg/ml) was added. Binding of sCD4 and C34-Ig was detected by fluorescein- and phycoerythrin-conjugated antibodies, respectively, as described in the Figure 3 legend. Note that the decrease in the mean fluorescence intensity of C34-Ig binding (right panel) was more modest than the decrease in the percentage of the double-positive cell population.
(0.44 MB TIF)

Figure S2 The kinetics, affinity, and consequences of soluble CD4 binding to the HIV-1 envelope glycoproteins. (A) Binding of sCD4 to the YU2 and AD8 envelope glycoprotein complexes on the surface of expressing cells was examined. COS-1 cells expressing the YU2 and AD8 full-length envelope glycoproteins were incubated with the indicated concentrations of sCD4 for 1 h at room temperature. Cells were then washed four times and incubated with the anti-CD4 monoclonal antibody OKT4 (10 µg/ml) for 30 min. Cells were subsequently washed, and binding was detected using a horseradish peroxidase-conjugated goat-anti-mouse polyclonal antibody. Results are presented as mean RLU (±s.e.m.) of two replicate samples. (B) The kinetics of CD4-Ig binding to full-length YU2 and JR-FL envelope glycoproteins expressed on the surface of COS-1 cells was measured by the cell-based ELISA method. CD4-Ig was added at 0.5 µg/ml. Results are presented as the percentage of binding measured at the final time point examined. (C) Decay of gp41 HR1 groove exposure for the indicated envelope glycoproteins at 25°C after pulse-activation with sCD4. (D) Decay of gp41 HR1 exposure at 25°C and 37°C after pulse activation by sCD4 (40 µg/ml; 0.8 µM). (E) Decay of HIV-1(KB9) and HIV-1(89.6) infectivity at 37°C after pulse activation by sCD4 (40 µg/ml; 0.8 µM).
(0.68 MB TIF)

Figure S3 Decay of HIV-1 infectivity at different temperatures after pulse activation with sCD4. (A) HIV-1(JR-FL) virions were magnetically immobilized on tissue-culture plates and pulsed with sCD4 (40 µg/ml; 0.8 µM) for three minutes at 26°C. Samples were then washed and incubated at the indicated temperatures for 20 min. Cf2Th-CCR5 cells were subsequently added and pelleted onto the viruses. Two days later, infectivity was measured by luciferase assays. (B) As a control, viruses were pulsed with buffer, incubated at the different temperatures, and then Cf2Th-CD4/CCR5 cells were added. Infectivity was measured two days later and is presented as mean RLU (±s.e.m.) of three replicate samples.
(0.31 MB TIF)

Figure S4 Decay of infectivity of viruses bearing different HIV-1 envelope glycoproteins. Recombinant, luciferase-expressing viruses that contain the indicated envelope glycoproteins were incubated in culture medium at 37°C for different time periods and then added to Cf2Th-CD4/CCR5 cells. 48 h later, luciferase activity in the target cells was measured to estimate the level of infection. Data are presented as the percentage of infectivity observed in samples not incubated at 37°C. The inset shows a comparison between the infectivity half-lives of the non-activated viruses (measured at 37°C with CD4+CCR5+ cells) and the sCD4-activated viruses (measured at 26°C with CD4−CCR5+ cells).
(0.43 MB TIF)

Figure S5 Change in cell-free HIV-1 infectivity after engagement of sCD4. HIV-1(YU2) or HIV-1(JR-FL) virions were associated with magnetite nanoparticles and then incubated with the indicated concentrations of sCD4 for different time periods at 26°C. Complexes were then added to confluent cultures of Cf2Th-CCR5 cells to which a magnetic field was applied. After incubation for 1 min, cells were washed and further cultured for two days. As a control, complexes of viruses and magnetite nanoparticles were incubated with buffer and then adsorbed to Cf2Th-CD4/CCR5 cells. Results are presented as mean RLU (±s.e.m.) of three replicate samples.
(0.66 MB TIF)

Figure S6 Activation of the HIV-1 envelope glycoproteins by cell-surface CD4. (A) The abilities of the YU2 and YU2-GS8 envelope glycoproteins to bind different ligands were compared, using the cell-based ELISA. COS-1 cells transfected with plasmids expressing the indicated envelope glycoproteins were incubated with the monoclonal antibodies 39F or IgG1b12 (both at 0.5 µg/ml), or C34-Ig (20 µg/ml), with or without sCD4 (20 µg/ml; 0.4 µM). Data are presented as mean RLU (±s.e.m.) of two replicate samples. (B,C) 293T cells transfected with a plasmid expressing wild-type human CD4 or with the control ΔKS plasmid were added to COS-1 cells transfected with plasmids expressing ΔKS, wild-type YU2, or the YU2-GS8 envelope glycoproteins. Cells were pelleted to increase approximation and incubated at 4°C for 75 minutes to allow CD4 engagement. Samples were then incubated with C34-Ig (20 µg/ml) to measure HR1 groove exposure (B) or the anti-CD4 antibody sc-70660 (Santa Cruz Biotechnology) to measure association of the 293T-CD4 cells with the COS-1 cell monolayer. Antibody binding was detected using HRP-conjugated antibodies. Data are presented as mean RLU (±s.e.m.) of two replicate samples. (D) The experiments described in (B) were repeated, expressing CD4 and the HIV-1 YU2 envelope glycoproteins (or the ΔKS control) in either COS-1 or 293T cells.
(0.56 MB TIF)

Figure S7 Relationship between the amount of transfected CCR5-expressing plasmid DNA and the density of CCR5 on the surface of the transfected cells. (A) COS-1 cells cultured in 96-well plates (2.4×10^4^ cells per well) were transfected using Effectene reagent (Qiagen) with the indicated amounts of the pcDNA3.1-CCR5 plasmid, which expresses the CCR5 gene under the control of the hCMV-IE promoter. Each well was transfected with the same total amount of DNA (0.1 µg) by supplementing the pcDNA3.1-CCR5 plasmid with the ΔKS plasmid. Two days later, CCR5 expression was measured using the cell-based ELISA method with the anti-CCR5 monoclonal antibody 2D7 (1 µg/ml), detected with a horseradish peroxidase-conjugated goat-anti-mouse IgG2a antibody. Data are presented as mean RLU (±s.e.m.) of two replicate samples. (B) Cf2Th cells cultured in 10-cm plates were transfected using Lipofectamine 2000 reagent (Invitrogen) with the indicated amounts of the pcDNA3.1-CCR5 plasmid. Three days later, cells were analyzed for CCR5 expression with a phycoerythrin-conjugated 2D7 antibody using an EPICS XL flow cytometer (Beckman-Coulter). Data were analyzed using WinMDI 2.9 software.
(0.18 MB TIF)
