# Supplementary figures and images for: Soluble CD4 and CD4-Mimetic Compounds Inhibit HIV-1 Infection by Induction of a Short-Lived Activated State
Source: PLoS Pathog. 2009 Apr 3;5(4):e1000360. doi: 10.1371/journal.ppat.1000360 (PMC2655723; doi:10.1371/journal.ppat.1000360)

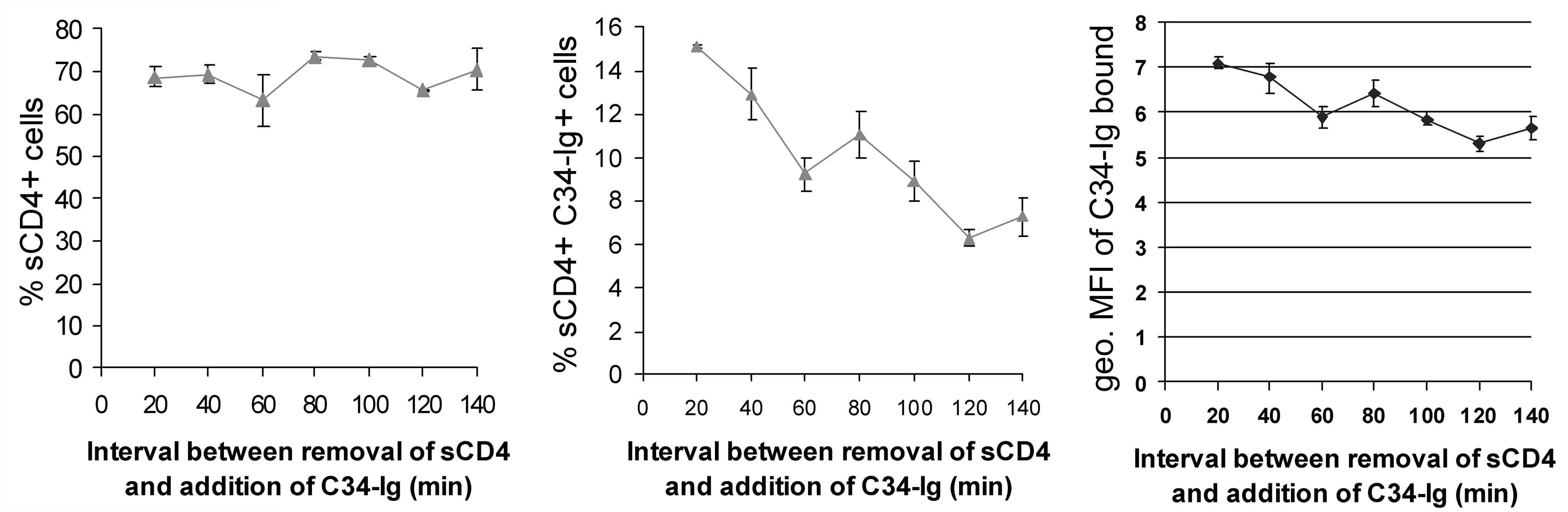

Supplement: Figure S1 — Decay of HR1 groove exposure measured by FACS. Cells that express the YU2(Δct) envelope glycoproteins were incubated at room temperature with sCD4 (40 µg/ml; 0.8 µM) for 20 min, washed, and further incubated at room temperature for the indicated time periods. Then the C34-Ig protein (40 µg/ml) was added. Binding of sCD4 and C34-Ig was detected by fluorescein- and phycoerythrin-conjugated antibodies, respectively, as described in the Figure 3 legend. Note that the decrease in the mean fluorescence intensity of C34-Ig binding (right panel) was more modest than the decrease in the percentage of the double-positive cell population. (0.44 MB TIF) [file ppat.1000360.s001.tif]

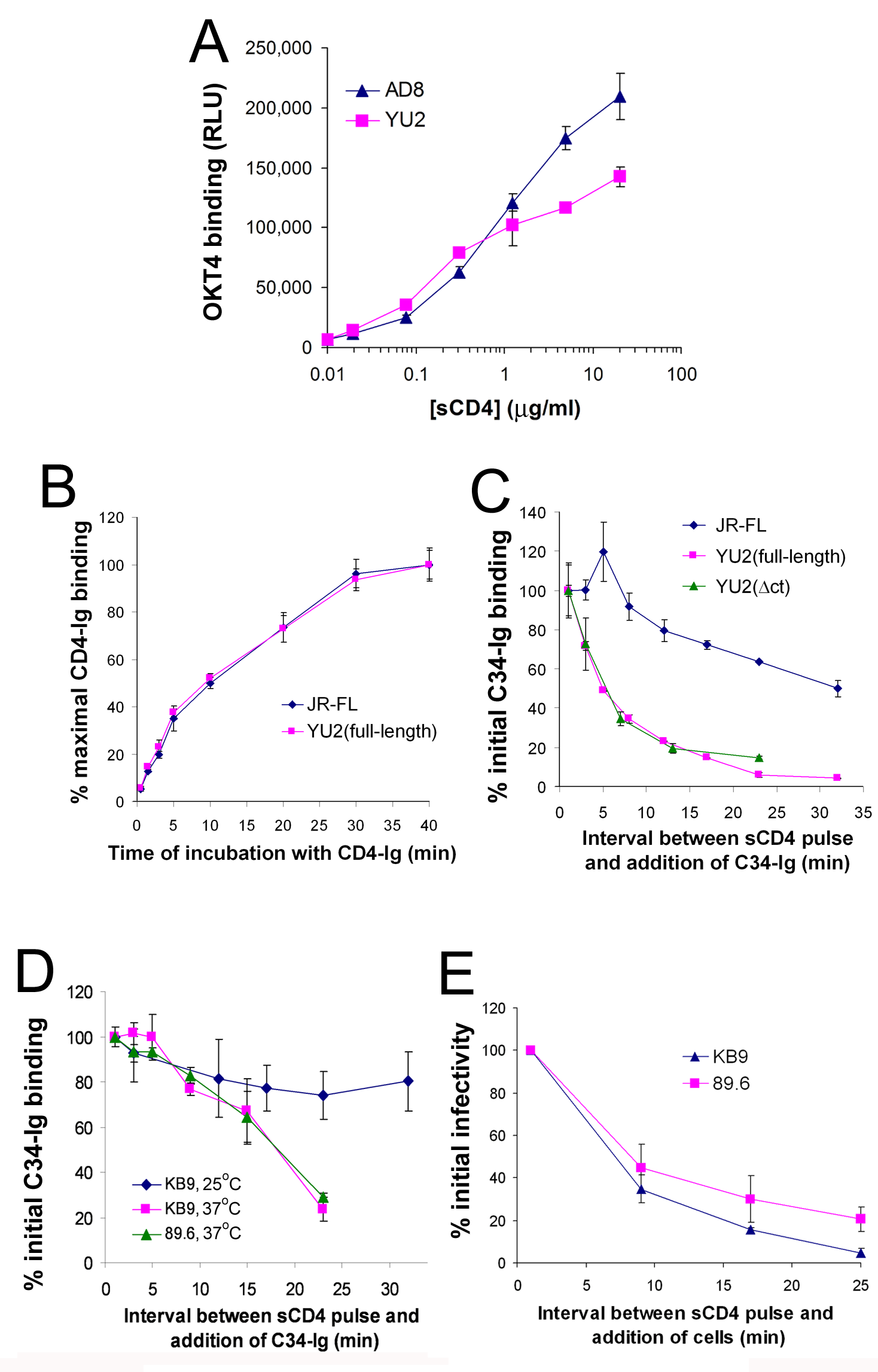

Supplement: Figure S2 — The kinetics, affinity, and consequences of soluble CD4 binding to the HIV-1 envelope glycoproteins. (A) Binding of sCD4 to the YU2 and AD8 envelope glycoprotein complexes on the surface of expressing cells was examined. COS-1 cells expressing the YU2 and AD8 full-length envelope glycoproteins were incubated with the indicated concentrations of sCD4 for 1 h at room temperature. Cells were then washed four times and incubated with the anti-CD4 monoclonal antibody OKT4 (10 µg/ml) for 30 min. Cells were subsequently washed, and binding was detected using a horseradish peroxidase-conjugated goat-anti-mouse polyclonal antibody. Results are presented as mean RLU (±s.e.m.) of two replicate samples. (B) The kinetics of CD4-Ig binding to full-length YU2 and JR-FL envelope glycoproteins expressed on the surface of COS-1 cells was measured by the cell-based ELISA method. CD4-Ig was added at 0.5 µg/ml. Results are presented as the percentage of binding measured at the final time point examined. (C) Decay of gp41 HR1 groove exposure for the indicated envelope glycoproteins at 25°C after pulse-activation with sCD4. (D) Decay of gp41 HR1 exposure at 25°C and 37°C after pulse activation by sCD4 (40 µg/ml; 0.8 µM). (E) Decay of HIV-1(KB9) and HIV-1(89.6) infectivity at 37°C after pulse activation by sCD4 (40 µg/ml; 0.8 µM). (0.68 MB TIF) [file ppat.1000360.s002.tif]

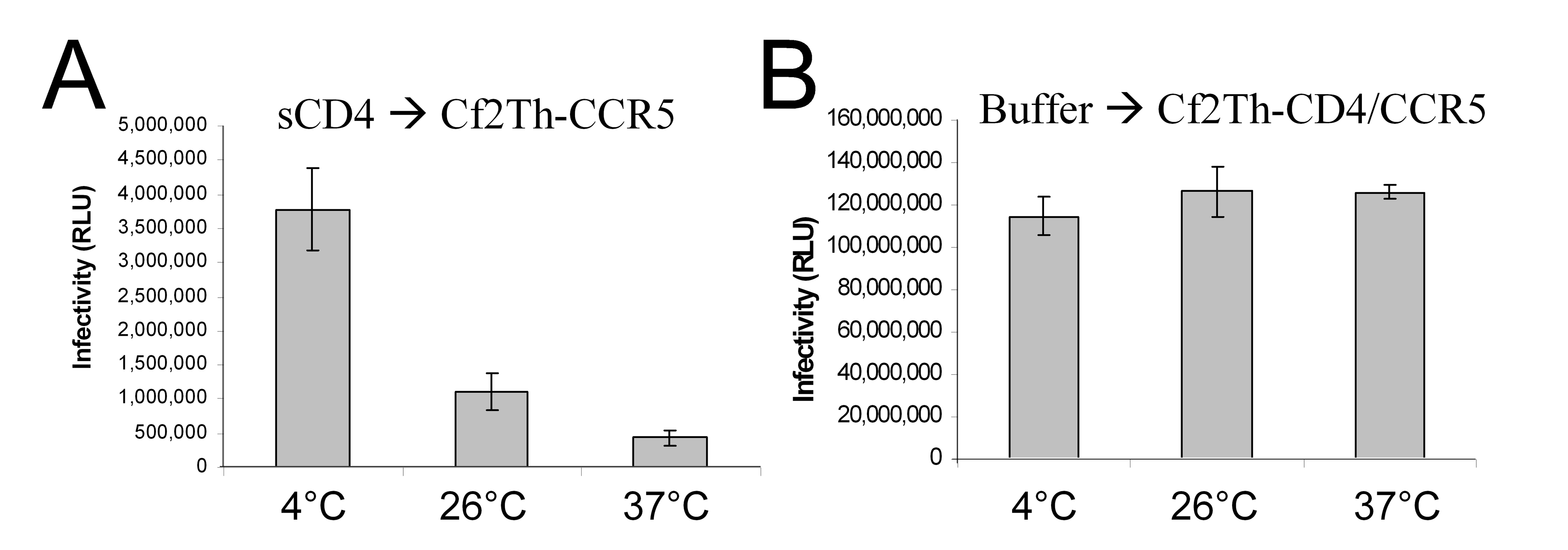

Supplement: Figure S3 — Decay of HIV-1 infectivity at different temperatures after pulse activation with sCD4. (A) HIV-1(JR-FL) virions were magnetically immobilized on tissue-culture plates and pulsed with sCD4 (40 µg/ml; 0.8 µM) for three minutes at 26°C. Samples were then washed and incubated at the indicated temperatures for 20 min. Cf2Th-CCR5 cells were subsequently added and pelleted onto the viruses. Two days later, infectivity was measured by luciferase assays. (B) As a control, viruses were pulsed with buffer, incubated at the different temperatures, and then Cf2Th-CD4/CCR5 cells were added. Infectivity was measured two days later and is presented as mean RLU (±s.e.m.) of three replicate samples. (0.31 MB TIF) [file ppat.1000360.s003.tif]

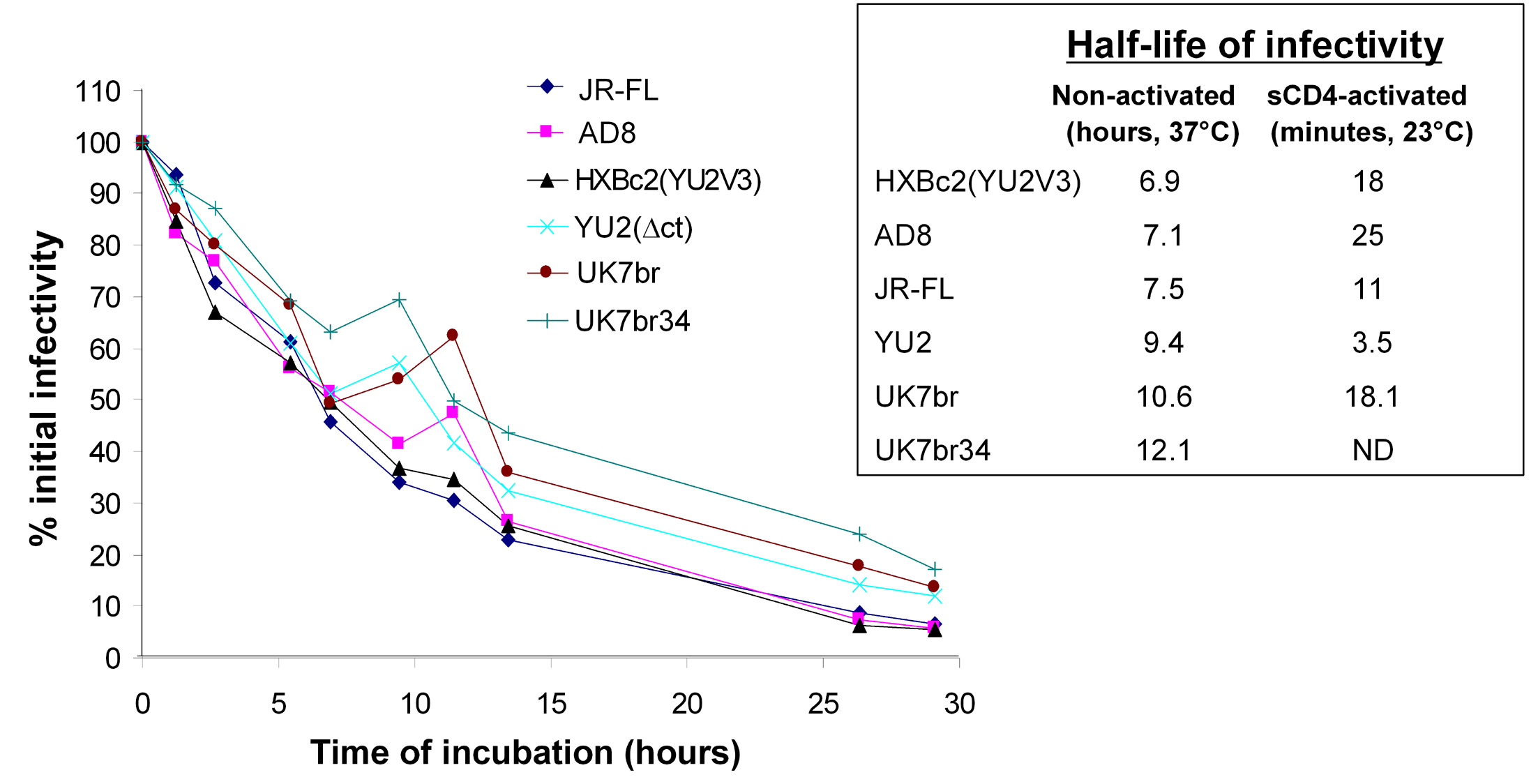

Supplement: Figure S4 — Decay of infectivity of viruses bearing different HIV-1 envelope glycoproteins. Recombinant, luciferase-expressing viruses that contain the indicated envelope glycoproteins were incubated in culture medium at 37°C for different time periods and then added to Cf2Th-CD4/CCR5 cells. 48 h later, luciferase activity in the target cells was measured to estimate the level of infection. Data are presented as the percentage of infectivity observed in samples not incubated at 37°C. The inset shows a comparison between the infectivity half-lives of the non-activated viruses (measured at 37°C with CD4+CCR5+ cells) and the sCD4-activated viruses (measured at 26°C with CD4−CCR5+ cells). (0.43 MB TIF) [file ppat.1000360.s004.tif]

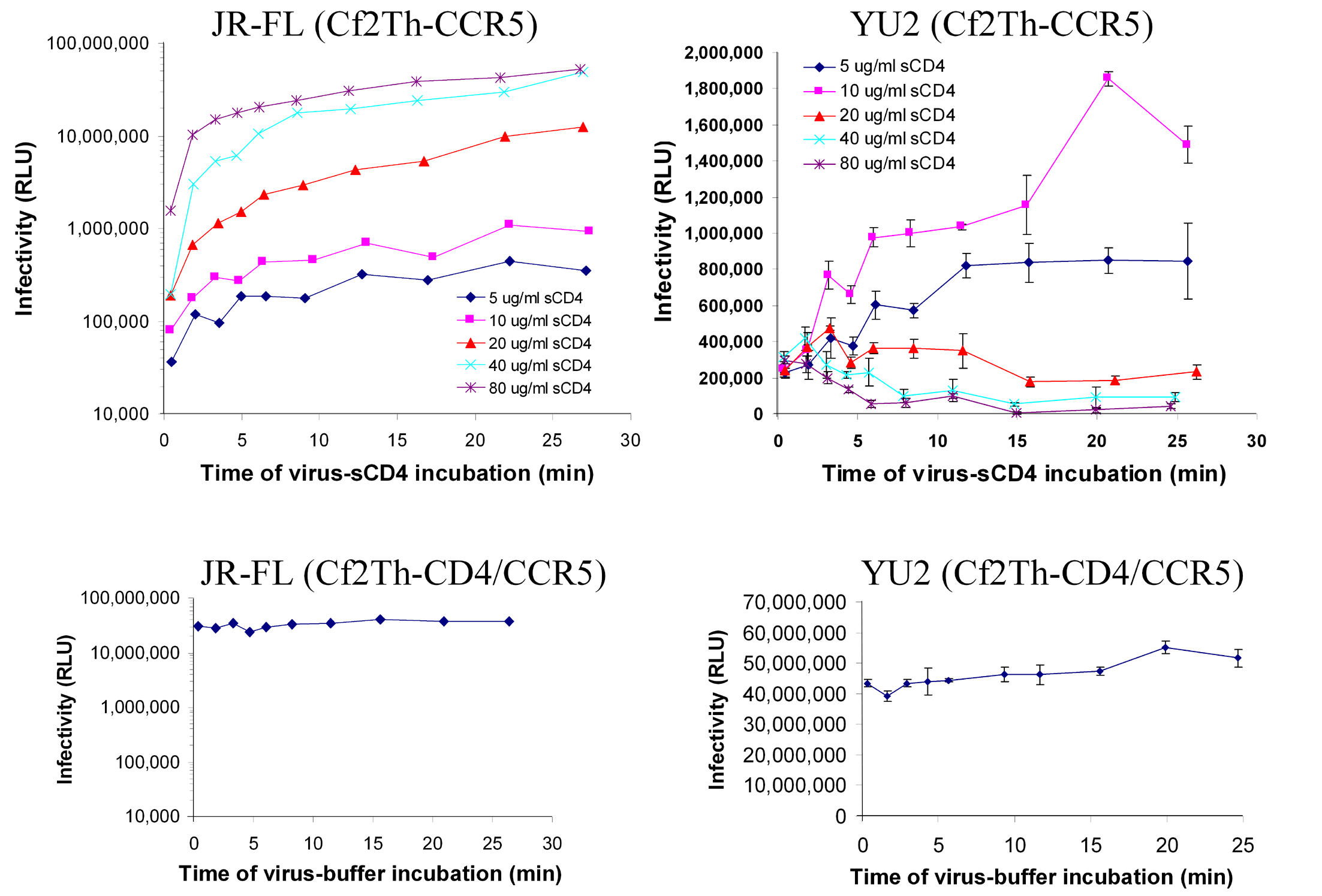

Supplement: Figure S5 — Change in cell-free HIV-1 infectivity after engagement of sCD4. HIV-1(YU2) or HIV-1(JR-FL) virions were associated with magnetite nanoparticles and then incubated with the indicated concentrations of sCD4 for different time periods at 26°C. Complexes were then added to confluent cultures of Cf2Th-CCR5 cells to which a magnetic field was applied. After incubation for 1 min, cells were washed and further cultured for two days. As a control, complexes of viruses and magnetite nanoparticles were incubated with buffer and then adsorbed to Cf2Th-CD4/CCR5 cells. Results are presented as mean RLU (±s.e.m.) of three replicate samples. (0.66 MB TIF) [file ppat.1000360.s005.tif]

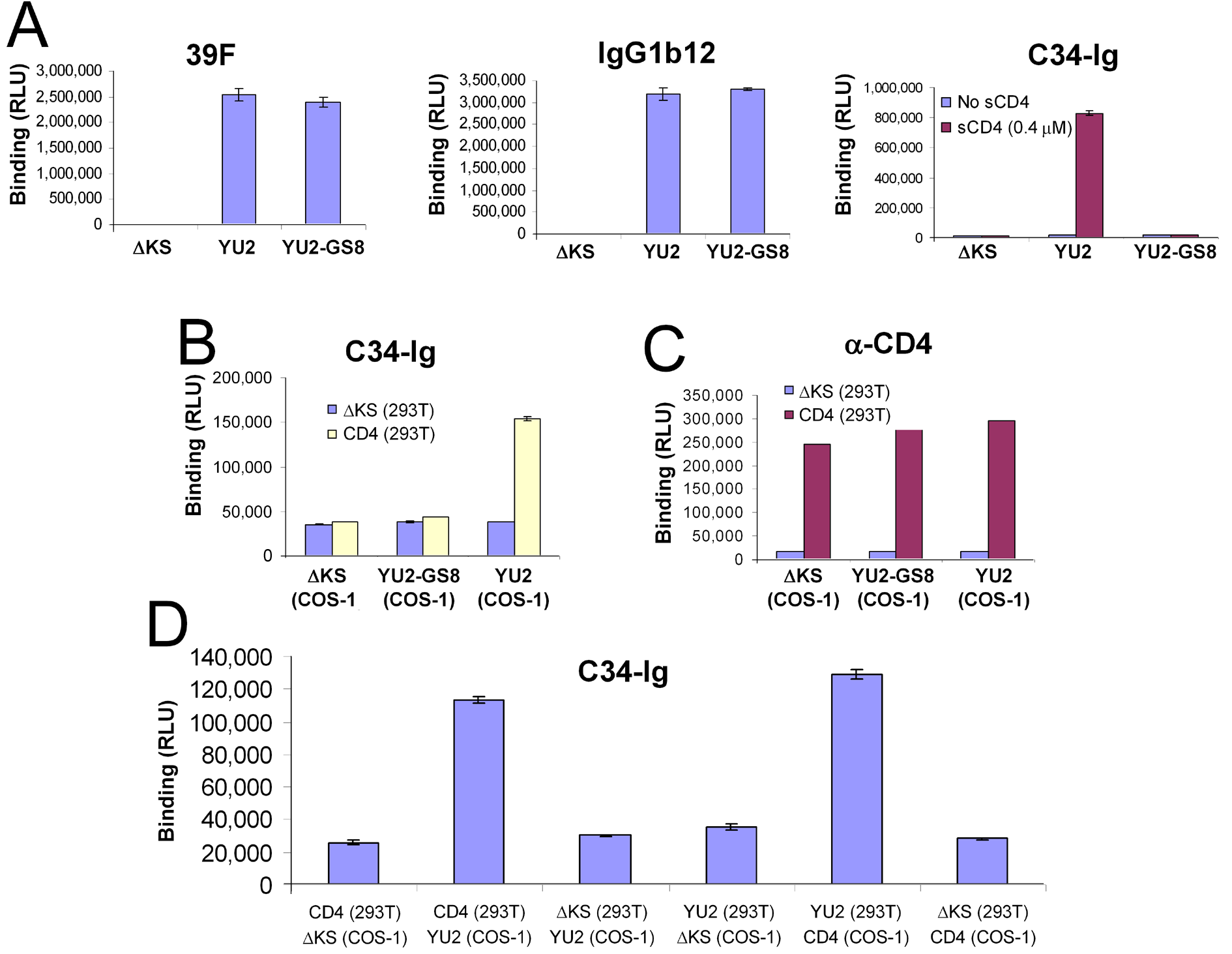

Supplement: Figure S6 — Activation of the HIV-1 envelope glycoproteins by cell-surface CD4. (A) The abilities of the YU2 and YU2-GS8 envelope glycoproteins to bind different ligands were compared, using the cell-based ELISA. COS-1 cells transfected with plasmids expressing the indicated envelope glycoproteins were incubated with the monoclonal antibodies 39F or IgG1b12 (both at 0.5 µg/ml), or C34-Ig (20 µg/ml), with or without sCD4 (20 µg/ml; 0.4 µM). Data are presented as mean RLU (±s.e.m.) of two replicate samples. (B,C) 293T cells transfected with a plasmid expressing wild-type human CD4 or with the control ΔKS plasmid were added to COS-1 cells transfected with plasmids expressing ΔKS, wild-type YU2, or the YU2-GS8 envelope glycoproteins. Cells were pelleted to increase approximation and incubated at 4°C for 75 minutes to allow CD4 engagement. Samples were then incubated with C34-Ig (20 µg/ml) to measure HR1 groove exposure (B) or the anti-CD4 antibody sc-70660 (Santa Cruz Biotechnology) to measure association of the 293T-CD4 cells with the COS-1 cell monolayer. Antibody binding was detected using HRP-conjugated antibodies. Data are presented as mean RLU (±s.e.m.) of two replicate samples. (D) The experiments described in (B) were repeated, expressing CD4 and the HIV-1 YU2 envelope glycoproteins (or the ΔKS control) in either COS-1 or 293T cells. (0.56 MB TIF) [file ppat.1000360.s006.tif]

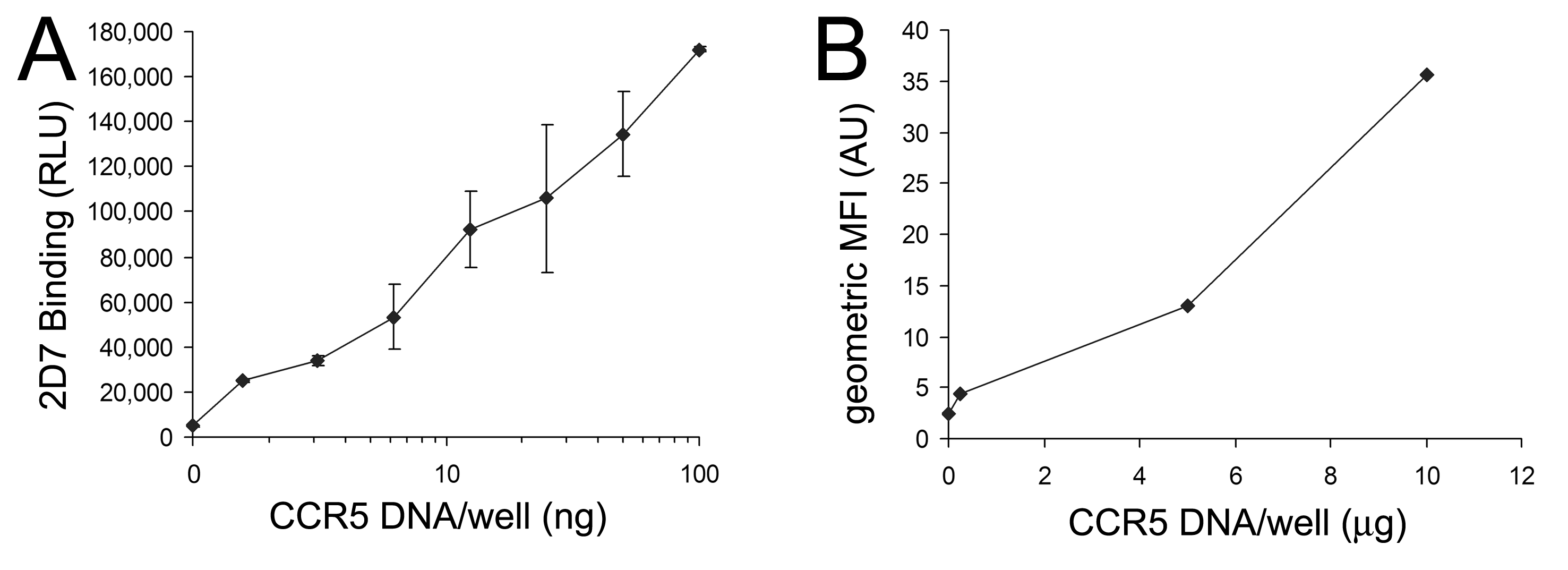

Supplement: Figure S7 — Relationship between the amount of transfected CCR5-expressing plasmid DNA and the density of CCR5 on the surface of the transfected cells. (A) COS-1 cells cultured in 96-well plates (2.4×10^4^ cells per well) were transfected using Effectene reagent (Qiagen) with the indicated amounts of the pcDNA3.1-CCR5 plasmid, which expresses the CCR5 gene under the control of the hCMV-IE promoter. Each well was transfected with the same total amount of DNA (0.1 µg) by supplementing the pcDNA3.1-CCR5 plasmid with the ΔKS plasmid. Two days later, CCR5 expression was measured using the cell-based ELISA method with the anti-CCR5 monoclonal antibody 2D7 (1 µg/ml), detected with a horseradish peroxidase-conjugated goat-anti-mouse IgG2a antibody. Data are presented as mean RLU (±s.e.m.) of two replicate samples. (B) Cf2Th cells cultured in 10-cm plates were transfected using Lipofectamine 2000 reagent (Invitrogen) with the indicated amounts of the pcDNA3.1-CCR5 plasmid. Three days later, cells were analyzed for CCR5 expression with a phycoerythrin-conjugated 2D7 antibody using an EPICS XL flow cytometer (Beckman-Coulter). Data were analyzed using WinMDI 2.9 software. (0.18 MB TIF) [file ppat.1000360.s007.tif]
